# Evaluation of Biological Activity of *Polygala Boliviensis* in Experimental Models

**DOI:** 10.22037/ijpr.2019.1100655

**Published:** 2019

**Authors:** José Luiz Carneiro da Rocha, Renata Freiras Araujo de Tripodi Calumby, Danielle Figueredo da Silva, Hugo Neves Brandão, Cristiane Flora Villarreal, Flávia Oliveira de Lima

**Affiliations:** a *Departamento de Saúde, Universidade Estadual de Feira de Santana, Feira de Santana, Bahia, Brasil.*; b *Faculdade de Farmácia, Universidade Federal da Bahia, Salvador, Bahia, Brasil. *

**Keywords:** Polygalaceae, Analgesics, Pain, Natural product, Antiedematogenic

## Abstract

The plants of the genus *Polygala* (Polygalaceae) are employed in folk medicine for the treatment of several pathologies, including disorders of the bowel and kidney, as anesthetic, expectorant and anti-inflammatory. The present study was undertaken to investigate the antiedematogenic and antinociceptive activities of methanolic extract of *Polygala boliviensis *A. W. Benn (MEPB) in mice. The antinociceptive activity of MEPB was evaluated using the writhing, formalin, and tail immersion tests. The carrageenan-induced paw edema test was used to assess the antiedematogenic activity of MEPB. Mice motor performance was evaluated in the rota rod and open field tests and the acute toxicity were evaluated over 14 days. High-performance liquid chromatography was used to determine the fingerprint chromatogram of MEPB. Oral administration of MEPB (75- 600 mg/kg) reduced the number of writhing induced by acetic acid. In the formalin test, the oral pre-treatment with MEPB (75 - 600 mg/kg) produced a dose-related inhibition only of the late phase. MEPB (300 and 600 mg/kg) reduced the carrageenan-induced paw edema. In contrast, the treatment with MEPB (300 and 600 mg/kg) did not prevent the thermal nociception in the tail immersion test. MEPB (600 mg/kg)-treated mice did not show any motor performance alterations. Over the study duration of 14 days, there were no mortality or toxic signs recorded in the group mice given 6000 mg/kg of MEPB. The present study demonstrated, for the first time, the antinociceptive and antiedematogenic properties of *Polygala boliviensis*.

## Introduction

Pain is a major health problem that limits productivity and reduces quality of life. So, the analgesics represent a therapeutic class with high consumption in worldwide. However, the current analgesic, such as opioids and nonsteroidal anti-inflammatory drugs, are not useful in many cases because of their adverse effects and a significant number of patients obtain no relief from these treatments (1; 2). Thus, the search for new agents that have potent analgesic activity and limited adverse effects is of great interest.

The natural products may serve as the leads and scaffolds for elaboration of efficacious drugs for a several diseases. The natural products and/ or natural product structures have important role in discovery of many clinically important drugs in the current therapy. However, many sources of natural products remain largely untapped (3; 4). 

Polygalaceae family comprises 19 genera and approximately 1300 species, distributed in almost all parts of world, especially in neotropic areas (5; 6). Among the members of this family, the *Polygala* is the most representative genus (7). Species from the genus *Polygala* have been used in folk medicine to treat several diseases, including bronchitis, neurasthenia, inflammation, amnesia, and as topical anaesthetic and expectorant (8; 9). Moreover, many *Polygala* species are well-known for producing several classes of secondary metabolites and a variety of pharmacological effects, such as trypanocidal (10), antiviral (11), hypoglycaemic (12), anti-tumoural (13; 14; 15), neuroprotective (16; 17), anti-inflammatory (18; 19; 20), antinociceptive (21; 22; 23), and antipsychotic activities (24).

*Polygala boliviensis*, popularly known as “arrozinho”, is a small herb (18-35 cm long) growing in the Bolivia and Brazil (25). It is used in folk medicine as a diuretic, expectorant, against blennorrheas, and to treat snake bites. Apart from these medicinal uses, there is a report indicating its antibacterial/antibiofilm properties (26). Thus, the present study was undertaken to investigate the antiedematogenic and antinociceptive activity of methanolic extract of *Polygala boliviensis* A. W. Benn (MEPB).

## Experimental

*Plant Material*

*Polygala boliviensis* A. W. Benn (PB) collected in September 2010 during the morning period from campus of the University of Feira de Santana (UEFS) was identified by José Floriano Barea Pastore. A voucher specimen (HUEFS 2687) was deposited at the Herbarium of the Department of Botany, University of Feira de Santana.

*Extraction*

The aerial parts of plant dried in a forced-air oven at 45 ± 3 °C, powered (240 g), soaked in 0, 8 L of MeOH for 5 weeks (weekly renovation). The solvent was removed under reduced pressure on a rotary evaporator at 60 °C, resulting in the crude extract (41,14 g, yield: 17, 14%).

*Phytochemical screening and HPLC-DAD analysis*

To know the composition of extract, the colorimetric reactions or precipitation were used. The phytochemical screening was conducted as described previously (27). The presence of alkaloids was determined with Dragendorff’s and Mayer’s reagents; saponins by foam test; flavonoids by Shinoda test; phenols were measured with ferric chloride; steroids and terpenoids by Liebermann-Burchard reaction; coumarins by lamp UV after alkalinization of the extract; tannins with lead acetate and copper acetate; antraquinones by Borntraeger reaction and anthocyanins by exposure to different pHs.

The analysis of the extract was performed with high pressure liquid chromatography on a Hitachi model EZChrom Elite HPLC system equipped with a VRW HITACHI L-2130 pump, a VRW HITACHI L-2300 oven, a VRW HITACHI L-2455 Diode-Array Detector (DAD) coupled with an auto injector. The extract was analyzed using a reverse-phase HPLC column: LiChroCART Purospher Star® RP18-e (250 mm x 4,6 mm i.d., 5µm) (Merck, Darmastad, Germany) coupled with LiChroCART 4-4 LiChrospher 100RP18 (5µm) (Merck, Darmastad, Germany). The mobile phase consisted of acetonitrile (A) and water (B) using a gradient of 50% A for 0-5 min, 50-60% A for 5-15 min, 60-90% A for 15-18 and 90-50% A for 18-22 min. The column temperature was maintained at 25 ºC with a flow rate of 0, 7-1mL/min. An injection volume of 20 μL of the extract was used. The sample was prepared by taking an aliquot of the methanol extract and dissolving in hexane to give a concentration of 10 mg / mL. Then, 1 mL of hexane solution was subjected to filtration cartridge Solid Phase Extraction (Solid-Phase Extraction - SPE) with 4 mL of HPLC grade acetonitrile. Approximately, 1.5mL of the acetonitrile solution obtained was again filtered through microporous membrane (0.22 mM) directly to a vial, which was subjected to chromatography for identification and quantification of the compound of interest. Spectral data were recorded in 236 nm throughout the entire procedure.

*Animals*

The experiments were performed using male Swiss mice (22-28 g) obtained from the Animal Facilities of the University of Feira de Santana. The animals were housed in temperature-controlled rooms (22-25 ºC), under a 12:12 h light-dark cycle, with access to water and food *ad libitum* until use. All experiments were approved by the Animal Experimentation Ethics Committee of UEFS (004/2012) and performed in accordance with the guidelines of the International Association for Study of Pain (IASP) on the use of animals in pain research (28). All behavioral tests were performed between 7:00 AM and 5:00 PM, and the animals were only used once.

*Writhing Test*

The mice were treated with MEPB (75, 150, 300 and 600 mg/kg) or vehicle (saline; negative control) by the oral route 60 min before acetic acid injection. Indomethacin (10 mg/kg; i.p.) was used as the positive control 30 min before acetic acid injection. Acetic acid (0.8% v/v, 10 mL/kg) was injected into the peritoneal cavities of mice, which were placed in a large glass cylinder, and the intensity of nociceptive behavior was quantified by counting the total number of writhes occurring between 0 and 30 min after the stimulus injection. The writhing response consists of a contraction of the abdominal muscle together with a stretching of the hind limbs (29). Antinociceptive activity was expressed as the writhing scores over 30 min.

*Formalin Test*

The mice were treated with MEPB (75, 150, 300 and 600 mg/kg) or vehicle (saline; negative control) by oral route 60 min before injection under the surface of the right hind paw of 20 μL of 2.5% formalin (1:100 dilution of stock formalin solution, 37% formaldehyde in 0.9% saline). Indomethacin (10 mg/kg; i.p.) was used as the positive control 30 min before formalin injection. After formalin injection, the mice were observed from 0 to 05 min (early phase) and from 15 to 30 min (late phase), and the nociception score was determined by counting the time that the animal spent licking the injected limb during the observation time (30).

*Tail immersion test*

The tail immersion was performed as previously described (31). Before the day of the experiment, each animal was habituated to the restraint cylinder for 4 consecutive days (20 min per day). On the experimental day, the mice were placed in the restraint cylinder and the tail tip (2 cm) was immersed in a water bath at 48 °C ± 0.5 °C. The latency for the tail withdrawal reflex was measured before (baseline) and after treatments. To prevent tissue damage of the tail, the cut-off time was 10 s. Mice were then randomly selected to perform in one of experimental groups: control (saline, i.p.), MEPB (300 and 600 mg/kg, oral) and morphine (5 mg/kg, s.c.; reference drug) and tested for antinociception at 0.5, 1, 3, and 5 h following the administration of the drugs. Antinociception of each mouse was calculated according to the following formula: Antinociception index = [(test latency – baseline latency) / (10- baseline latency)] x100.

*Paw Edema Test*

The mice were treated with MEPB (150, 300 and 600 mg/kg) or vehicle (saline; negative control) by the oral route 60 min before carrageenan injection (100 μg/paw). Dexamethasone (2 mg/kg, s.c.) was used as the positive control 40 min before carrageenan injection. The volume of the mice paws was measured with a plesthismometer (Ugo Basile, Comerio, Italy) before (V_O_) the intraplantar injection with carrageenan and 3 h after (V_T_), as described previously (32). The amount of paw swelling was determined for each mouse and the difference between V_T_ and V_O_ was taken as the edema value (mm^3^/paw).

**Figure 1:**
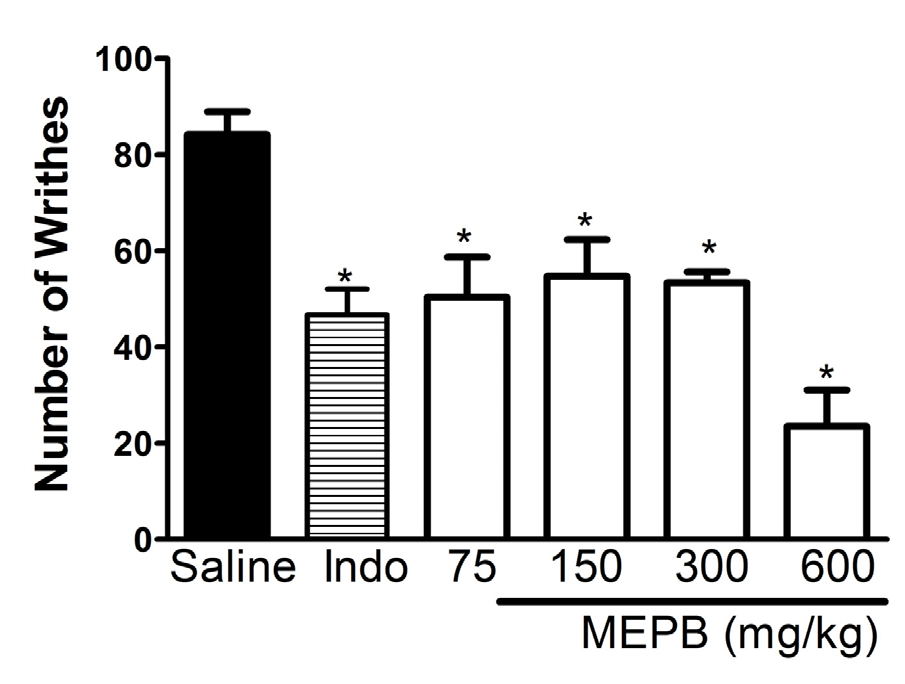
Effects of oral administration of MEPB on acid acetic-induced writing response. Mice were treated with MEPB (75-600 mg/ kg, oral) or saline (control group) 1 h before acetic acid. Indomethacin (Indo; 10mg/kg, i.p.) was the reference drug administered 30 min before the acid acetic. Results are presented as means± SEM of 6 mice per group. *Significantly different from control group (*p *< 0.05), ANOVA followed by Bonferroni’s test.

**Figure 2:**
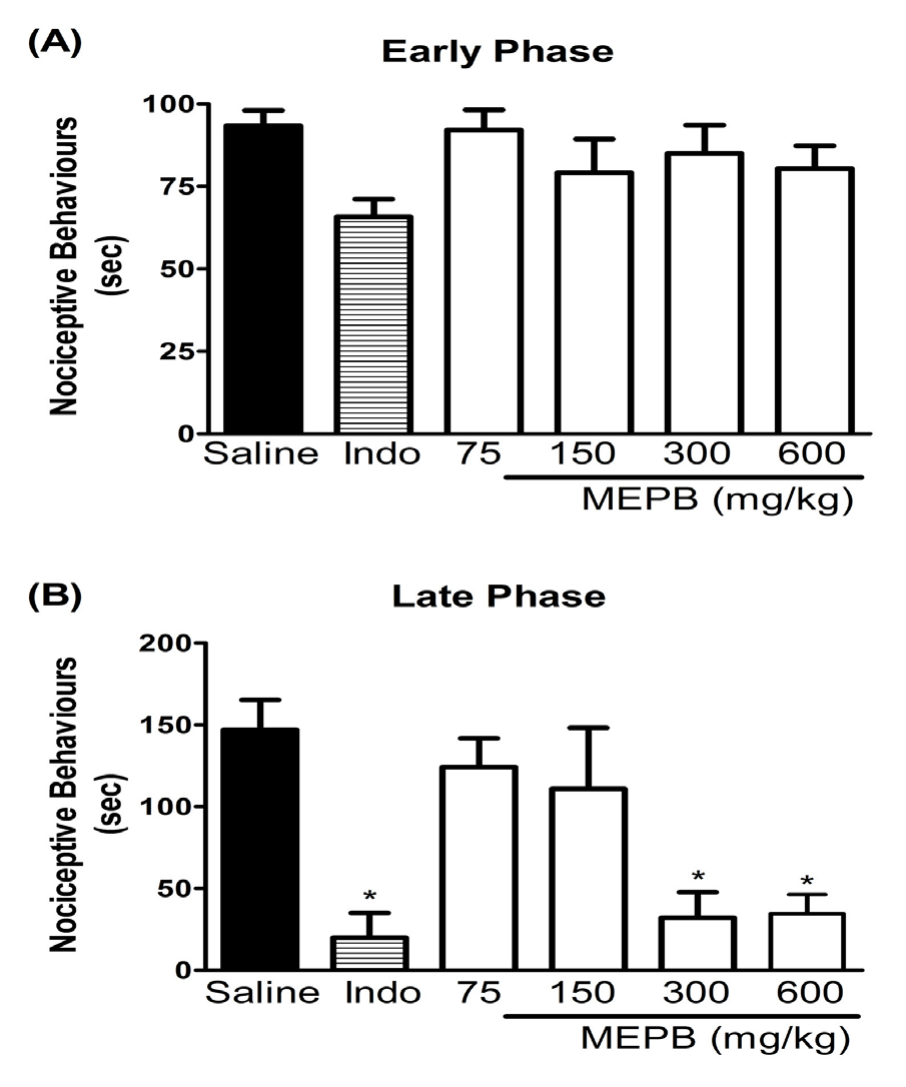
Effects of oral administration of MEPB on the formalin test**. **Panels (A) and (B) represent effects of MEPB on the neurogenic and inflammatory phases of formalin test, respectively. Mice were treated with MEPB (75-600 mg/kg, oral) or saline (control group) 1 h before of the formalin injection. Indomethacin (Indo; 10 mg/kg, i.p.) was the reference drug administered 30 min before the formalin injection. Results are presented as means± SEM of 6-7 mice per group. *Significantly different from control group (*p *< 0.05), ANOVA followed by Bonferroni’s test

**Figure 3:**
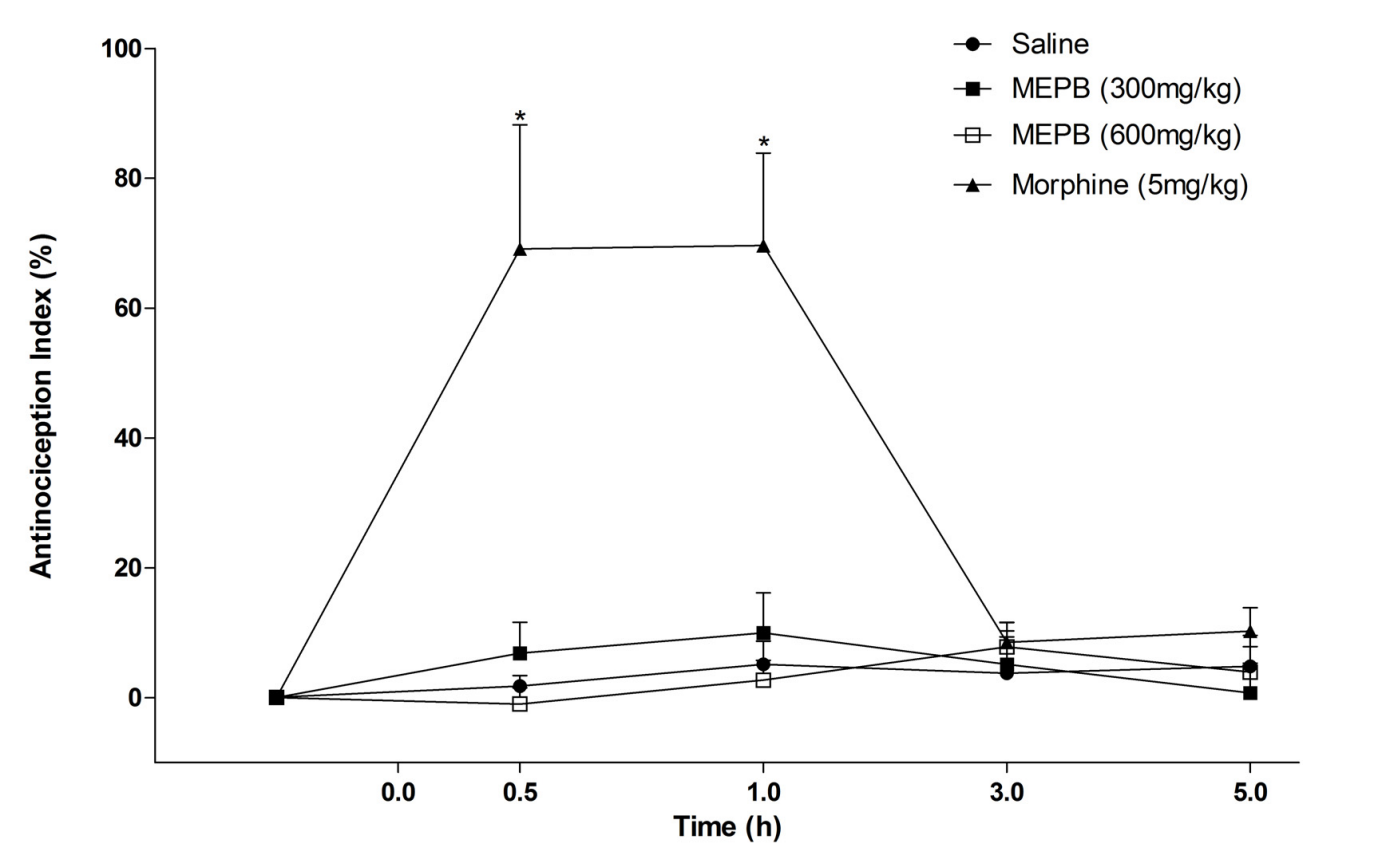
Effects of oral administration of MEPB on tail immersion test. The Figure show data of tail withdrawal reflex latencies represented as antinociception%. Thermal nociceptive threshold was evaluated before and up 5 h following administration of MEPB (300 and 600 mg/kg) or saline. Morphine (5 mg/kg, s.c) was the reference drug. Data are expressed as means ± S.E.M.; n = 6 mice per group. *Significantly different from control group (*p *< 0.001), Two-way ANOVA followed by the Bonferroni’s test

**Figure 4:**
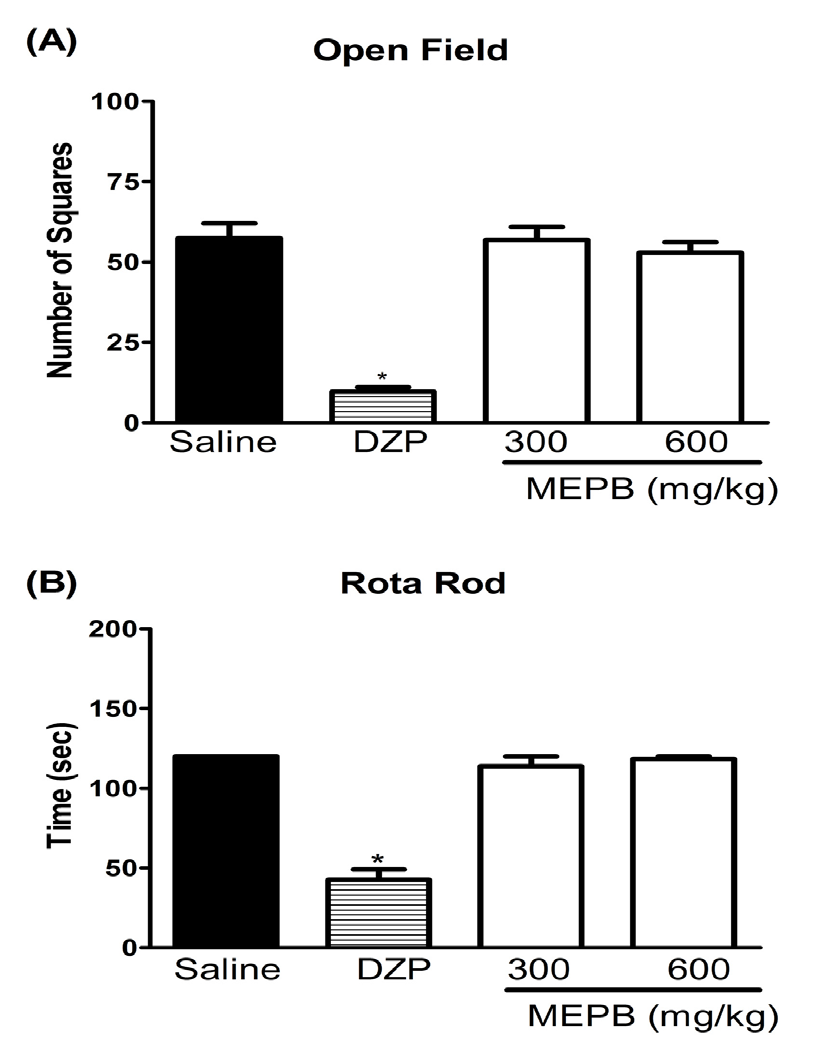
Effects of oral administration of MEPB on motor function. Panels (A) the number of square crossings in the open field and (B) the run time on the rota rod, 1 h after the oral administration of MEPB (600 mg/kg) or saline. Diazepam (DZP, 10 mg/kg, i.p.) was the reference drug administered 30 min before testing. Data are expressed as means ± S.E.M.; n = 6 mice per group. *Significantly different from control group (*p *< 0.001), ANOVA followed by Bonferroni’s test

**Figure 5:**
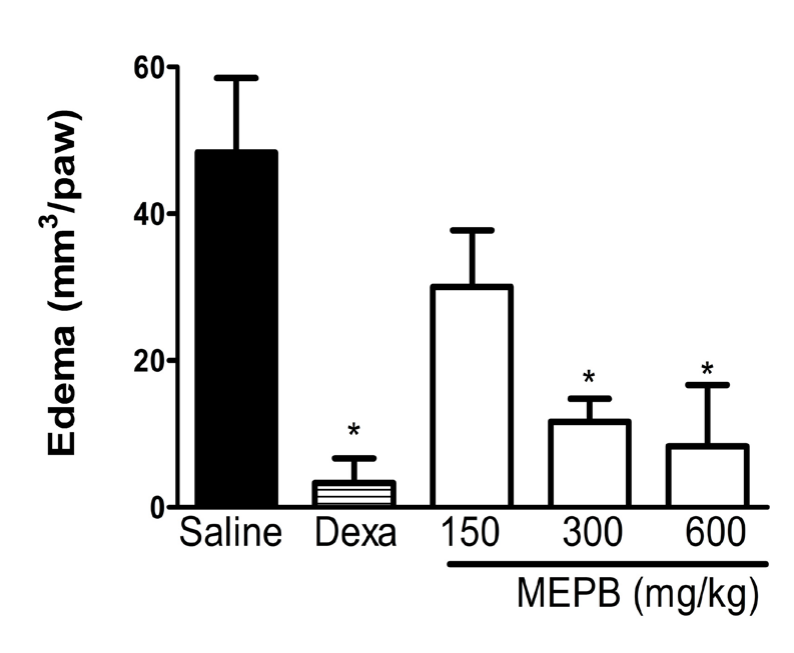
Effects of oral administration of MEPB on carrageenan-induced oedema paw. Mice were treated with MEPB (150- 600 mg/kg,oral) or saline (control group) 30 min before carrageenan. Dexamethasone (Dexa; 2mg/kg, s.c.) was the reference drug. Results are presented as means ± SEM of 6 mice per group. *Significantly different from control group (*p*< 0.05), ANOVA followed by Bonferroni’s test

**Figure 6:**
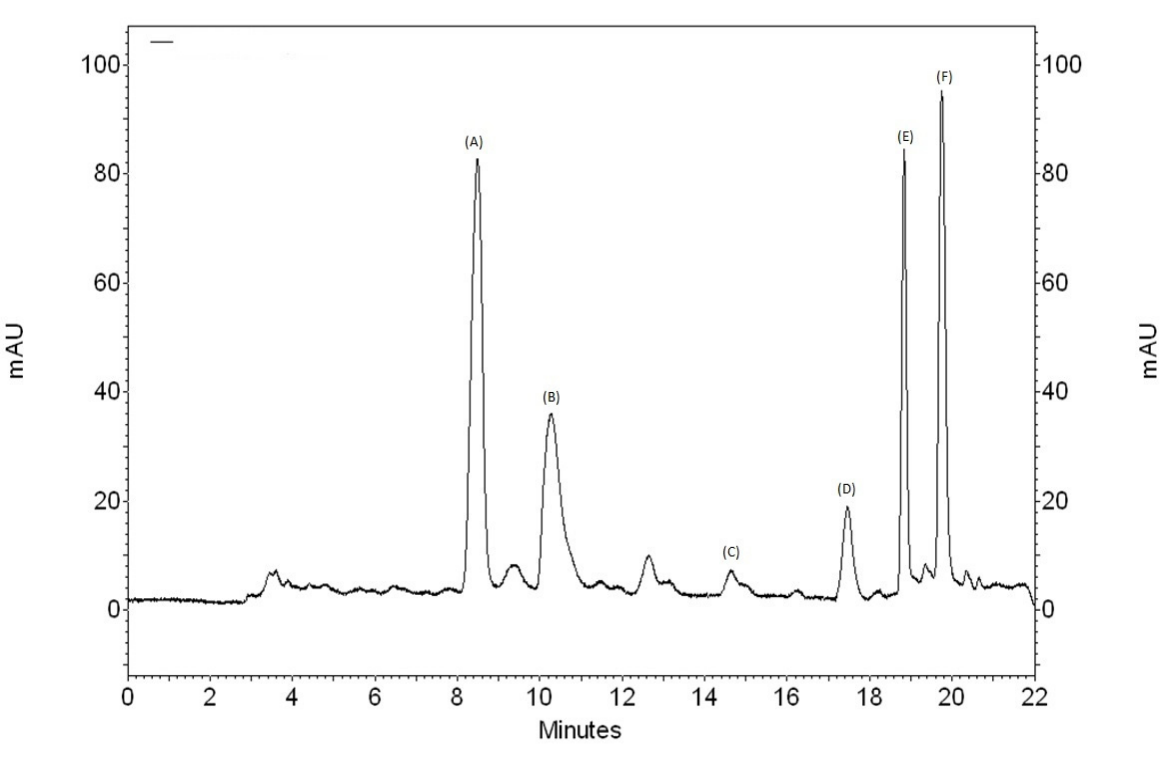
Chromatogram of the crude methanol extract *Polygala boliviensis*

**Figure 7:**
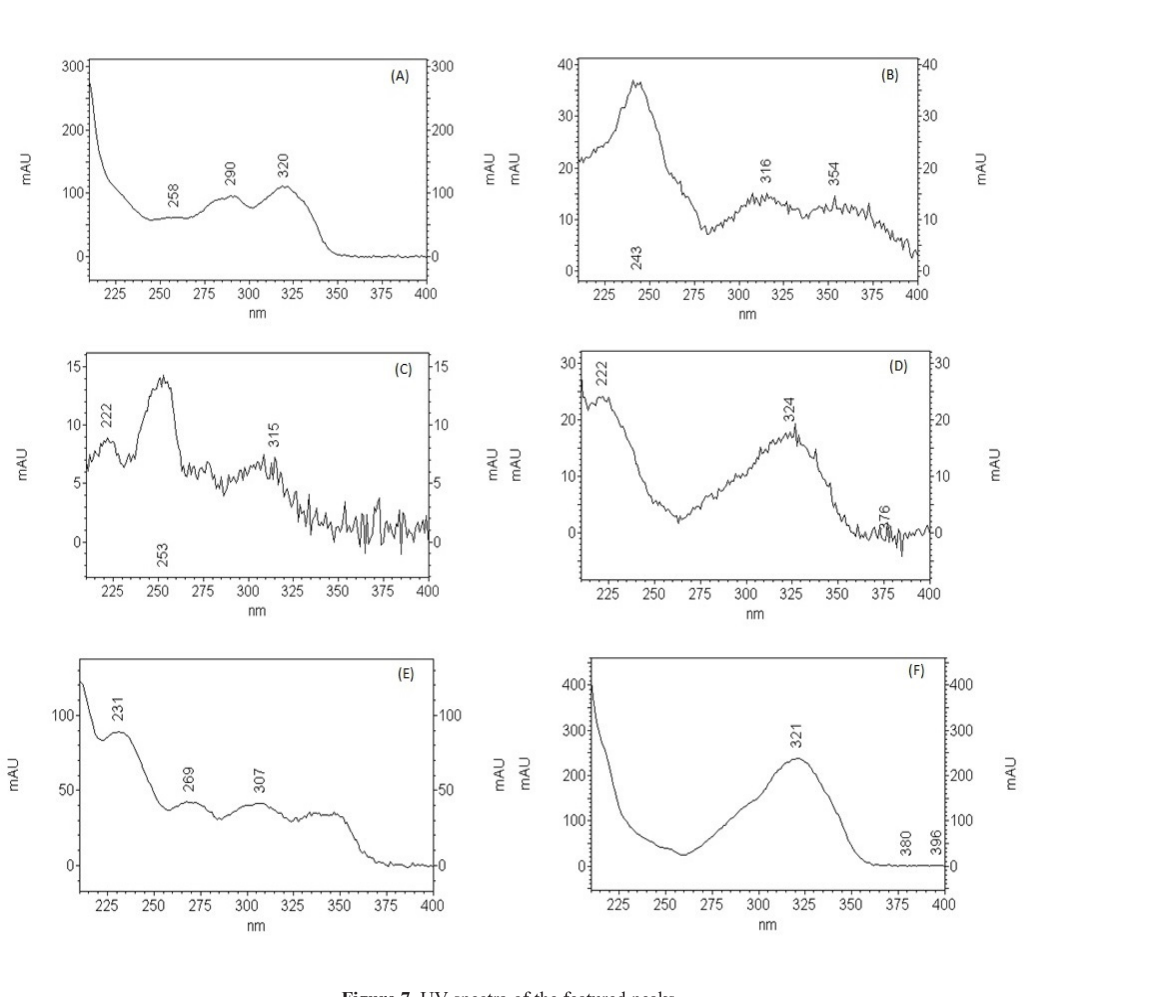
UV spectra of the featured peaks

*Rota-rod test*

Mice motor function was evaluated on the rota-rod test (33). The apparatus consisting of a bar with a diameter of 3 cm was subdivided into four compartments (Insight, Ribeirão Preto, Brazil). The bar was rotated at a constant speed of 16 rpm. The animals were select 24 h previously by eliminating those mice that did not remain on the bar for two consecutive periods of 120 s. The animals were treated with MEPB (300 and 600 mg) by oral route or vehicle (10 mg/kg, i.p.) 1 h after being placed on a rotating rod. Diazepam (10 mg/kg; i.p.) was used as the positive control. The cutoff time used was 120 s. The results are expressed as the average time (s) the animals remained on the rota-rod in each group.

*Open Field test*

Ambulatory behavior was assessed in an open field test. The mice were treated MEPB (300 and 600mg/kg) or saline (control group) and 1 h afterward they were placed individually in a wooden box (40x60x50 cm) with the floor divided into 12 squares. Diazepam (10 mg/kg; i.p.) was used as the positive control. The number of squares crossed with the four paws was measured for a period of 3 min.

*Acute toxicity test*

Acute oral toxicity test was conducted in accordance with Lorke (34), with slight modification. To investigate the potential toxicity of MEPB, the mice received a single oral of MEPB (600, 3000 and 6000 mg/kg) or vehicle (saline). Access to food and water, toxic symptoms and the general behavior of the mice were observed continuously for 1 h after the treatment, intermittently for 4 h, and thereafter over a period of 24 h. The mice were further observed for up to 14 days following treatment for any signs of toxicity and mortality.

*Drugs and dilutions*

All drugs used in this study were purchased from Sigma^®^ Chemical Co., St. Louis, MO, USA (Indomethacin, Dexamethasone and Carrageenan), Merck^®^ (Formaldehyde and Acetic acid) and Cristália^®^ (Morphine, Diazepam). Indomethacin was dissolved in Tris-HCl 0.1 M pH 8.0 plus physiological saline. The remaining drugs were dissolved in physiological saline.

*Data analysis*

The data are presented as means ± standard error of the mean (SEM). The differences between groups were evaluated by one-way ANOVA with Bonferroni’s post hoc test or repeated measures of two-way ANOVA with Bonferroni’s post-hoc test, when appropriate. Statistical differences were considered to be significant at *p* < 0.05.

## Results

*Antinociceptive Effect of MEPB*

The antinociceptive effect of MEPB was initially evaluated, using the writhing test in mice. Oral administration of MEPB (75 - 600mg/kg), 1h before acid acetic injection, produced a significant (*p* < 0.05) inhibition of writhing response in the mice (Figure.1). Indomethacin (10 mg/kg), a standard nonsteroidal anti-inflammatory drug (NSAID), used as reference drug, also produced a significant inhibition of writhing response (*p* < 0.05).

The formalin test was used to confirm the antinociceptive effect of MEPB. Injection of formalin in control mice induced a biphasic behavioral response, with the first phase ranging from 0 to 5 min (Figure. 2A) and the second phase from 15 to 30 min (Figure 2B) after injection. The treatment with MEPB (300 and 600 mg/kg) by oral route 1 h before formalin injection, caused antinociceptive effect only in the second phase of test (*p* < 0.05). Indomethacin (10 mg/kg), the reference drug, significantly inhibited the late phase of the formalin test (*p* < 0.05).

Other test conducted was the tail flick test used to evaluate a possible central antinociceptive effect. The treatment with MEPB (300 and 600 mg/kg) did not alter the latency response in the tail immersion test (Figure 3). In contrast, morphine (5 mg/kg), an opioid analgesic used as positive control, significantly increased the response latency at 0.5 and 1 h after administration (*p* < 0.001).

*Effect of MEPB on motor function*

The treatment with MEPB, at the therapeutic doses, did not affect the motor performance of the mice in the rota-rod and open field tests, indicating that the observed antinociception was unrelated to sedation or motor abnormality (Figure 4). In contrast, diazepam (10 mg/kg) treatment, used as positive control, significantly decreased the number of crossing on the open field test and the time on the rota rod test (*p* < 0.001).

*Antiedematogenic Effect of MEPB*

The antiedematogenic effect of MEPB was evaluated with the paw edema test induced by carrageenan (Figure 5). The treatment with MEPB (300 and 600 mg/kg) significantly reduced the carrageenan-induced paw edema (*p* < 0.05), confirming the antiedematogenic effect of this plant. Dexamethasone (2 mg/kg), used as reference drug, also produced a significant antiedematogenic effect (*p* < 0.05).

*Acute toxicity of MEPB*

Over the study duration of 14 days, the MEPB administration MEPB (600, 3000 and 6000 mg/kg) by oral route did not produce any variations in the general appearance, toxic signs or mortality, suggesting low toxicity.

*Phytochemical study and HPLC-DAD analysis*

The phytochemical study demonstrates that MEPB was positive for the presence of alkaloids, coumarins, saponins, flavonoids, phenols, tanins, steroids, and terpenoids. However, it was not possible to detect the presence of anthraquinones and anthocyanins.

In the HPLC analysis, the chromatogram (Figure 6) shows the presence of methyl salicylate (14.65 min) that was confirmed by comparison of retention time and UV band characteristic with standard, and other peaks that have characteristics of flavonoids and others phenolics compounds (Figure 7).

## Discussion

Some species of *Polygala* are employed in folk medicine worldwide. In Brazil, *Polygala boliviensis *has been used to treat snake bites, as a diuretic, expectorant and against blennorrheas (26). The present study demonstrates that oral administration of *Polygala boliviensis *extract, at doses that did not induce any motor performance alteration, produced consistent antinociceptive and antiedematogenic effects in different models of pain and inflammation. 

The antinociceptive effect of MEPB was firstly evaluated, using the writhing test in mice, a screening tool for assessment of the analgesic effect of new substances (29). Oral administration of MEPB produced a significant inhibition of writhing response in mice. In line with this result, the previous studies demonstrated antinociceptive action of others species from the genus *Polygala*, such as *Polygala cyparissias *(21), *Polygala sabulosa *(23), and *Polygala paniculata* (22). 

The writhing test is described as typical model of visceral inflammatory pain (29). This method has good sensitivity, but lacks specificity since non-analgesic compounds may inhibit the writhing response (35). Thus, the formalin test, a chemical model of nociception, was used to confirm the antinociceptive effect of MEPB. The formalin test, which affords a more specific response compared with the writhing test, promotes a biphasic behavioral reaction. The first phase, known as neurogenic pain, results from direct stimulation of nociceptors, whereas the second phase, named inflammatory pain, is caused by release of inflammatory mediators (30). In the present study, the treatment with MEPB caused antinociceptive effect only in the second phase of test. The pharmacologic control of pain is based on two main strategies: the drugs that prevent nociceptor sensitization, such as NSAIDS (36) and the drugs that blocked the nociception transmission, such as opioids analgesics (37). Opioids analgesics suppress both phases of formalin, whereas NSAIDS seem to suppress only second phase of formalin test (35). Considering the inhibitory property of MEPB on the second phase of formalin, it is possible to propose that its antinociceptive activity is due, at least in part, to an anti-inflammatory action, preventing nociceptor sensitization.

In line with this idea, MEPB did not prevent the nociception in the tail immersion test, which mainly identifies central analgesics (35). The tail immersion test is a thermal model of pain considered to be a spinal reflex, but could involve higher neural structures. The fact that the treatment with MEPB did not alter the latency response to the tail immersion reinforces the hypothesis that MEPB induces peripheral antinociception. Moreover, the treatment with MEPB, at similar range of doses used in the nociceptive tests, did not affect the motor performance of the mice in the rota-rod and open field tests. This result confirms the antinociceptive effect of MEPB suggested by the nociceptive tests.

The effects of MEPB were evaluated on the carrageenan-induced paw edema in mice. This model is widely used to test new anti-inflammatory drugs. The inflammation caused by intraplantar injection of carrageenan involves the release of several inflammatory mediators, which increased blood supply and accumulation of leukocytes, resulting in edema (38). The treatment with MEPB significantly reduced the carrageenan-induced paw edema, confirming the antiedematogenic effect of this plant. The MEPB administration by oral route did not produce any variations in the general appearance, toxic signs, or mortality at 6 g/kg, suggesting low toxicity. 

The presence of alkaloids, coumarins, saponins, flavonoids, phenols, tanins, steroids, and terpenoids in MEPB could be attributed to the observed antinociceptive and anti-inflammatory activities. Other studies carried out with different species of *Polygala, *such as *Polygala paniculata* (22),* Polygala cyparissias* (21), and *Polygala sabulosa*, (39) also demonstrated the presence of coumarins, xanthones, steroids, and flavonoids, corroborating the present results. According Meotti *et al. *(39), the antinociceptive and antiinflamatory properties of *Polygala sabulosa *were attributed to the presence of coumarins and flavonoids. Xanthones seems to be responsible, at least in part, for the antinociceptive properties reported for *Polygala cyparissias *(21). Klein-Júnior *et al*. (40) attributed the presence of compounds such as steroids and xanthones to antihyperalgesic activity of *P. cyparissias*. Lapa *et al* (22) report that rutin, phebalosin and aurapten as well as others compounds found in *P. paniculata*, might act synergically contributing to the potent antinociceptive action of *P. paniculata.* Triterpenoids saponins and flavones have reported in *P. japonica*, and theses saponins inhibited both phases of the edema (41). 

Another study quantified the presence of methyl salicylate in different species of *Polygala *(42). The methyl salicylate seems to be responsible for the characteristic odor for the essential oil extracted from roots of *Polygala cyparissias*, as well as for use in folk medicine as an analgesic and anti-inflammatory preparation (9). In other study, methyl salicylate (89.1% and 97.8%) was found as the main volatile constituent in both roots of the *P. paniculata* and *P. cyparissias*, respectively (43). 

## Conclusion

In conclusion, this study demonstrates, for the first time, that the oral treatment with MEPB produces antinociceptive and antiedematogenic effects. Moreover, it indicates that MEPB did not affect the motor performance and it had a low toxicity at doses pharmacologically active. 
